# Towards a Uniform Metrological Assessment of Grating-Based Optical Fiber Sensors: From Refractometers to Biosensors

**DOI:** 10.3390/bios7020023

**Published:** 2017-06-21

**Authors:** Francesco Chiavaioli, Carlos A. J. Gouveia, Pedro A. S. Jorge, Francesco Baldini

**Affiliations:** 1 Institute of Applied Physics “Nello Carrara”, IFAC-CNR, Via Madonna del Piano 10, 50019 Sesto Fiorentino, Italy; f.baldini@ifac.cnr.it; 2 Institute for Systems and Computer Engineering, Technology and Science, INESC-TEC, Rua do Campo Alegre 687, Porto 4169-007, Portugal; icarlos07@gmail.com (C.A.J.G.); pedro.jorge@inesctec.pt (P.A.S.J.); 3 Institute INESC P&D Brasil, Rua José Caballero, 15, Gonzaga, 11055-300 Santos, Brazil

**Keywords:** optical fiber grating, fiber Bragg grating, long period grating, resonance-based device, refractive index sensor, biosensor, sensitivity, resolution, limit of detection, specificity

## Abstract

A metrological assessment of grating-based optical fiber sensors is proposed with the aim of providing an objective evaluation of the performance of this sensor category. Attention was focused on the most common parameters, used to describe the performance of both optical refractometers and biosensors, which encompassed sensitivity, with a distinction between volume or bulk sensitivity and surface sensitivity, resolution, response time, limit of detection, specificity (or selectivity), reusability (or regenerability) and some other parameters of generic interest, such as measurement uncertainty, accuracy, precision, stability, drift, repeatability and reproducibility. Clearly, the concepts discussed here can also be applied to any resonance-based sensor, thus providing the basis for an easier and direct performance comparison of a great number of sensors published in the literature up to now. In addition, common mistakes present in the literature made for the evaluation of sensor performance are highlighted, and lastly a uniform performance assessment is discussed and provided. Finally, some design strategies will be proposed to develop a grating-based optical fiber sensing scheme with improved performance.

## 1. Introduction

During the past twenty years but especially in the last ten, optical fiber grating (OFG) sensors have achieved wide interest both in fundamental research and for several industrial applications. In the last ten years, the number of publications in the field has reached almost 20,000, thus testifying the continuous growth and development of optical technology platforms based on OFGs.

These kinds of sensors have clearly their own advantages and drawbacks. As concerns the advantages, thanks to the peculiarity of optical fibers, we can mention the small physical size and weight that make OFG sensors suitable for the massive-scale production of both integrated optics and optoelectronic apparatuses, the immunity to both electromagnetic and radiofrequency interferences, the high compatibility with optoelectronic components/devices, the suitability of their use in hostile or harsh environments due to intrinsic electrical and chemical passivity, multiplexing and the remote measurement capabilities since the signal is spectrally modulated. This last feature is quite important, because it also implies that the readout is not affected by changes in the optical power caused by either fiber bending or source fluctuations. For these reasons, OFG sensors can attain superior metrological performance when compared with different technology-based platforms. On the other hand, OFG sensors have the major disadvantage of being sensitive to different measurands at the same time, such as temperature, refractive index (RI) of the medium surrounding the fiber, axial deformation (i.e., strain), pressure and humidity. This aspect entails the application of different strategies in order to make the device sensitive to the parameter of interest. In this work, the surrounding RI (SRI) will be the main parameter considered with an insight into the cross-sensitivity issue. Another drawback may derive from their fragility, thus requiring ad-hoc developed packaging or protections, especially for industrial applications.

However, given the increasing attention of the scientific community to OFG sensors, the need for a worldwide acceptable standardization of the sensing performance of an OFG sensor could be of general interest for both the research and industrial communities. The authors do not have the pretension of providing the “one and only” way of assessing the performance of an optical sensor. Instead, the aim of the present work is to offer a contribution based on both our knowledge and experience and on the literature published up to now, so as to provide a reference to facilitate any comparisons, not only within the same class of OFG sensors, but also among different kinds of sensors based on spectral resonance. The most significant parameters for a sensor are defined and described, along with the importance of denoting some crucial parameters with their correct names in order to be able to use the same parameters for describing the sensor performance. In addition, the most common and repeated mistakes in the literature, which arise from the great variety in the formulation and interpretation of the said parameters, are highlighted and discussed in detail.

One of the first clear and targeted attempts to provide “some basic definitions of sensor properties” to the scientific community in a standardized manner was made by D’Amico and Di Natale in 2001 [1]. In it, sensor response curve, sensitivity, noise, drift, resolution and selectivity are analyzed as the most frequently used parameters associated with sensor performance. The authors fittingly said of these features, “These words, if well-interpreted, represent a powerful vehicle of information and may symbolize part of a common knowledge useful for a sound dissemination of results relative to the sensor research.” After this, other papers were published that provided guidelines specifically designed for chemical and biochemical resonant sensors [2,3,4,5]. In 2008, White and Fan [2] focused on the explanation of the limit of detection (LOD) and the influence of the quality factor (Q-factor) for resonant RI sensors. Our major criticism of this paper is related to the term “LOD”, since in our opinion this could be misleading if related to refractometric sensors, in which it is more appropriate to talk about resolution, while its use fits perfectly for biosensors or, overall, for any sensor in which an interaction or binding with a target take place. In the same year, Janiga et al. [3] emphasized the important difference, for a chemical sensor or a biosensor, between the minimum detectable concentration (MDC) used by the International Organization for Standardization (ISO) and the LOD used by the International Union of Pure and Applied Chemistry (IUPAC). Both can be used, and represent compelling features, in assessing sensor performance. The key is simple: when considering the definitions, they both clearly state what the sensor is able to measure. In 2009, Hu et al. [4] proposed some “design guidelines for optical resonator biochemical sensors”. They appropriately pointed out the relevance of having a widely accepted figure of merit (FOM) with which to compare different technology platforms and, in this context, they focused on the Q-factor and RI LOD that strongly depend on the resonance peak full width at half maximum (FWHM). As in ref. [2], when someone wants to deal with the minimum RI change discernible from the noise that the sensor is able to measure, they should simply talk about sensor resolution in order to avoid an inexact usage of LOD. However, they also highlighted the influence of thermal fluctuations on sensor performance and the importance of their both having a good fitting procedure in order to reliably evaluate the resonance shift and to increase the number of measurements so as to reduce the noise contribution. All those features turn out to be crucial for improving sensor performance. More recently (2012), Loock and Wentzell [5] focused on the LOD evaluation for chemical and biochemical sensors, and reported the results obtained by comparing three different approaches for determining the detection limit: (i) an analysis of the standard deviations at low concentration; (ii) an evaluation of the instrumental resolution limit and (iii) an examination of the calibration curve. They concluded that the most appropriate way to evaluate the LOD is not to involve the sensor sensitivity and resolution, which in any case are two other important parameters. Rather, it should be determined either from the standard deviations at a low concentration by repeated measurements near the suspected LOD, as also suggested by the American Chemical Society (ACS) [6], or be calculated using the calibration curve, provided that the calibration points can be considered repeatable and reproducible.

The first attempt to give a metrological standardization focused only on OFG sensors was provided by Possetti et al. in 2012 [7]. They discussed in detail a method for evaluating uncertainties of measurement, applying it to both the FBG as a temperature sensor and the LPG as an RI sensor. The main conclusion was that, in OFG sensors, the major source of uncertainty (see Section 3.1.1) is related to repeatability (see Section 3.1.4) and reproducibility (see Section 3.1.5). This is ascribed to the combined effect of OFG cross-sensitivity and of environmental conditions, thus requiring some compensation procedures as well as an increase in the number of measurements.

The present work attempts to review critically and, at the same time, to combine all the assertions contained in all the papers published previously in the field, with the aim of providing a proper definition of the metrological parameters for the two main classes with regard to optical fiber sensors: the optical refractometers in which the measurement of SRI changes involves all the volume surrounding the sensor (in this case, we should talk about volume or bulk RI measurements) and the optical biosensors in which the measurement of SRI changes involves only the surface of the sensor on which the interaction with the target takes place (in this other case, one should talk about surface RI measurements). This distinction is particularly crucial in small volume analysis [8], as will be pointed out in Section 2. To be more precise, the work is divided as follows: Section 2 illustrates the fundamentals of OFGs focusing on both fiber Bragg gratings (FBGs) and long period gratings (LPGs); Section 3 defines the metrological parameters useful for assessing sensor performance, with some demonstrative examples of the most common mistakes and error present in the literature; Section 4 deals with the most challenging literature on OFG-based refractometers, whereas Section 5 details the same but as related to OFG-based biosensors with a detailed analysis of the cross-sensitivities’ issue of OFG sensors in the last two sections; Section 6 highlights some common mistakes present in the literature for the evaluation of OFG-based sensors and makes an attempt to provide a uniform procedure for assessing sensor performance. Lastly, Section 7 provides an outlook on the future perspective of OFG-based sensors.

## 2. Fundamentals of Optical Fiber Gratings

An OFG is a diffraction structure characterized by a periodic modulation of the RI within the core of a single-mode fiber (SMF), which satisfies the phase matching condition between the fundamental mode and other modes, either the core mode or the cladding modes or radiation (or leaky) modes [9]. Thanks to this phase matching, the fiber grating makes possible a controlled and efficient power transfer between modes within the optical fiber leading to a modulation of the transmitted spectrum. Depending on the range of its grating period Λ, OFGs can be classified into short period gratings, better known as FBGs, and into LPGs.

**Figure 1:**
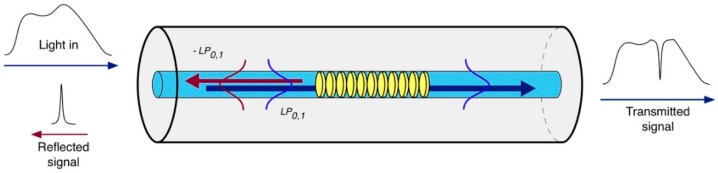
Sketch of a standard uniform fiber Bragg grating and its coupling mechanism.

The grating period of an FBG is typically of the order of hundreds of nm, resulting in a device that satisfies the phase matching between the fundamental core mode and its respective counter-propagating mode. Therefore, when a broadband optical signal reaches the grating, a narrow spectral fraction is reflected and the remaining is transmitted. The resonance wavelength $\lambda_{\mathrm{res}}$ at which the light is reflected satisfies the well-known Bragg condition [9]:

(1) $$\lambda_{\mathrm{res}}=2\;n_{\mathrm{core}}^{\mathrm{eff}}\;\Lambda ,$$

where $n_{\mathrm{core}}^{\mathrm{eff}}$ is effective RI of the core mode. The spectral width of the resonance peak is of the order of few hundreds of picometers, depending on the physical length of the grating. Figure 1 illustrates the principle of operation of an FBG.

**Figure 2:**
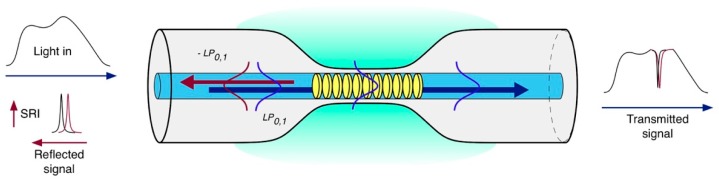
Sketch of an etched fiber Bragg grating and its coupling mechanism.

Standard uniform FBGs are intrinsically sensitive to temperature and strain [9], while they are not sensitive to the external RI due to the fact that the radiation is well confined within the fiber core. The first strategy used to overcome this issue involves a partial or total removal of the fiber cladding through etching [10], polishing processes [11] or by writing FBGs directly in microfibers [12]. Figure 2 illustrates the operating principle of an etched FBG (EFBG). The sensitivity of those devices is greatly dependent on the diameter of the fiber in the region of the grating. The lower the fiber diameter, the higher the sensitivity. Nevertheless, this process introduces fragility into the fiber sensor, especially when maximum sensitivity is required.

**Figure 3:**
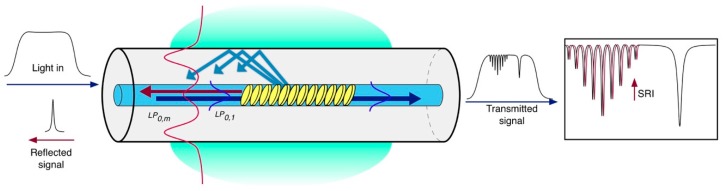
Sketch of a tilted fiber Bragg grating and its coupling mechanism.

The second approach relies on the intrinsic SRI sensitivity of the cladding modes excited by a tilted FBG (TFBG) [13], where the index modulation pattern is constantly tilted by an angle θ with respect to the fiber axis that makes possible the coupling of the propagating core mode to the cladding modes [14] as shown in Figure 3. In the case of the TFBG, the Bragg condition given in Equation (1) has to be rewritten by taking into account the resonance wavelength λ_res (m)_ of each m-th cladding mode, as follows [13]:

(2) $$\lambda_{\mathrm{res}\;\left(m\right)}=\left(n_{\mathrm{core}}^{\mathrm{eff}}-n_{\mathrm{clad}\;\left(m\right)}^{\mathrm{eff}}\right)\frac{\Lambda }{\cos\theta },$$

where θ is the tilt angle of the grating and $n_{\mathrm{clad}\;\left(m\right)}^{\mathrm{eff}}$ is the effective RI of the m-th cladding mode, which depends on the SRI. Typical tilt angles range between 4° and 16° [15].

In an LPG, the periodic modulation of RI usually ranges from 100 µm to 700 µm. This longer period leads to different phase matching conditions between the fundamental core mode and a discrete set of forward-propagating cladding modes (subscript m), in accordance with the following condition [16]:

(3) $$\lambda_{\mathrm{res}\;\left(m\right)}=\left(n_{\mathrm{core}}^{\mathrm{eff}}-n_{\mathrm{clad}\;\left(m\right)}^{\mathrm{eff}}\right)\Lambda .$$

**Figure 4:**
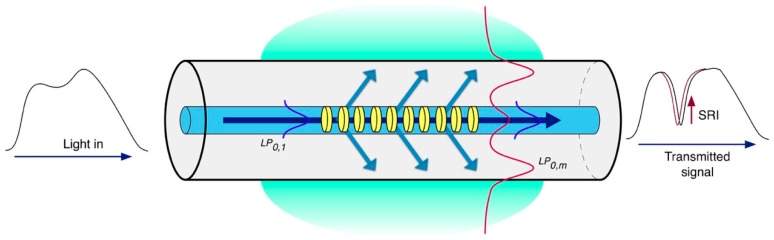
Sketch of a long period grating and its coupling mechanism.

The spectral width of the attenuation bands varies from only a few nanometers up to tens of nanometers, depending on the physical length of the grating, and this value increases with the order of the cladding mode [17]. Figure 4 illustrates the principle of operation of an LPG.

On the basis of Equations (2) and (3), it is clear how LPGs [18] and TFBGs [19] are intrinsically sensitive to the RI of the surrounding environment, making them highly suitable for chemical and biological sensing.

The next subsection gives special emphasis to RI sensing by addressing the crucial difference between a volume RI sensing and a surface RI sensing, which is often underestimated in many papers on the subject and hence deserves additional clarification.

### Refractive Index Sensing in Optical Fiber Gratings

**Figure 5:**
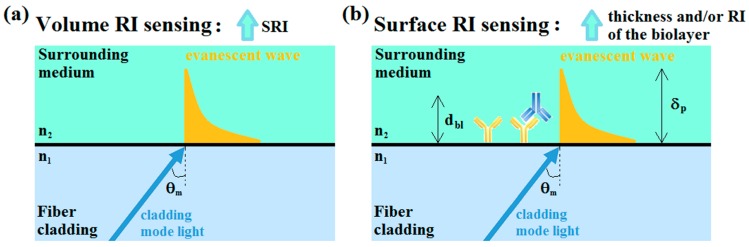
(**a**) Schematic representation of the RI sensing, based on a bulk or volume change; (**b**) schematic representation of the RI sensing, based on a surface change in which only a portion of the evanescent wave interacts with the bio-layer (d_bl_ < δ_p_). RI: refractive index; SRI: surrounding refractive index; n_1_: fiber cladding RI (or generally the RI of the denser medium); n_2_: surrounding medium RI (or generally the RI of the less dense medium); d_bl_: bio-layer thickness; δ_p_: evanescent field penetration depth.

OFG-based refractometers rely on the evanescent field interactions at the boundary between the fiber and the surrounding medium. The evanescent field occurs due to the interference between the incident and reflected rays, which generates an evanescent wave that propagates in the direction of the fiber axis and decays exponentially, perpendicular to the interface fiber/surrounding medium, as shown in Figure 5 [20]. The sensitivity of the OFG-based platforms is highly dependent on the capability of the evanescent field to penetrate the surrounding medium. Thus, the key parameter is the penetration depth δ_p_ (i.e., the distance from the interface at which the amplitude of the evanescent wave electric field decreases by a factor of 1/*e* in its exponential decay).

Generally, a larger δ_p_ implies a greater portion of radiation in contact with the surrounding medium, and hence a higher sensitivity [21]. Since δ_p_ depends on the coupled cladding mode as well as on the SRI and on the working wavelength, the longer the operation wavelength, the greater the penetration of the evanescent wave in the external medium will be [21]. In addition, higher order modes are less bound to the waveguide structure, extending further into the external medium (deeper penetration), and hence they are more sensitive to the SRI. In LPGs, since the order of the coupled mode is related to Λ, high cladding modes can be attained by decreasing the grating period [22]; while in TFBGs higher order modes are generated by using larger tilting angles [14].

As sketched in Figure 5, the difference between a bulk or volume RI change and an RI surface change deserves to be emphasized since it provides the essential distinction between an optical refractometer, which measures volume RI changes, and a biosensor, which measures surface RI changes that occur following the interaction between the bioanalyte to be measured and a sensing layer appropriately deposited on the fiber surface, hereinafter denoted as biolayer. Very recently, Sinibaldi et al. [23] pointed out a first preliminary distinction regarding this question, providing also an equation that relates the volume RI sensitivity to the surface sensitivity of the proposed biosensor.

In volume RI sensing (Figure 5a), the evanescent wave related to the coupled cladding mode (or in general the incident light) interacts with all the surrounding volume for the entire extent of its penetration depth. On the other hand, in surface RI sensing (Figure 5b), the interaction of interest is between the bio-layer and a portion of the evanescent wave, with the thickness of a bio-layer generally much less than the penetration depth of the evanescent wave (typically, tens of nm compared with 200–500 nm; i.e., d_bl_ < δ_p_). In this case, since the important measurand is constituted by RI changes induced along the sensor surface due to the interactions between the biological recognition element (BRE; usually a capture element) and the analyte under investigation, a washing step in a buffer solution is necessary after the injection of the analyte in order to measure only the shift in the resonance wavelength caused by the binding interaction [21]. A shift in the resonance wavelength (λ_res_) occurs, and can be measured in both cases, even if the wavelength shift related to a surface RI change is generally less than one related to a bulk RI change due to the molecular dimensions of the analyte interacting with the sensing layer [24,25].

For OFGs it is known that maximum sensitivity is achieved when the SRI is close to the cladding RI (n_sur_ ~ n_cl_), while for lower values of SRI (n_sur_ ~ 1.33–1.34 RIU, RI units), which is the range usually considered for biochemical approaches, these sensors are barely sensitive [22]. To overcome this limitation, the literature has accounted up to now for four different strategies for enhancing RI sensitivity. In the first one, the sensing portion of the device is coated with a thin layer of sub-wavelength thickness and with a higher RI (HRI) than the fiber cladding. This means that a relevant part of the optical power carried travels within the overlay. Depending on the thickness and RI of the overlay and on the SRI, this leads to a huge change in the propagation conditions of the optical signal [26], which extends its evanescent wave and results in an increased sensitivity to the SRI changes. Therefore, by carefully adjusting the overlay thickness it is possible to tune the maximum sensitivity to a desirable range of RI [27], similar to what is used for LPGs [27,28] and TFBG [29]. The second strategy concerns only LPGs, and consists of coupling the core mode to a high-order cladding mode that shows a turn-around point (TAP) in its phase-matching curve. In this case, the coupled cladding mode will show two attenuation bands that merge into a single broader band at its TAP. All the details can be found in the previous literature [30]. These special dual-band LPGs, better known as TAP LPGs [31], showed the greatest sensitivity among the LPGs manufactured on a bare fiber. Recently, by following the combination of the two previous approaches, it was theoretically demonstrated that TAP LPGs working in modal transition can reach a sensitivity of the order of 10^5^ nm·RIU^−1^, which is indubitably the highest one reported so far for fiber optic devices [32]. From an experimental point of view, a RI sensitivity of 1.2 × 10^4^ nm·RIU^−1^ was recently obtained in a narrow RI range that went from 1.3344 RIU to 1.3355 RIU [33].

A different way of enhancing the intensity of the evanescent field at the interface between two transparent materials is to combine OFGs with surface plasmon resonance (SPR) [34]. Here, fiber gratings covered with a thin metallic layer are used as an alternative optical platform to excite SPR due to their core-cladding efficient coupling mechanism and physical robustness, especially in the case of TFBGs [35] and LPG [36]. Lastly, the interest in OFGs written in microstructured optical fibers (MOF) for sensing has grown considerably [37,38,39]. In fact, these fibers show unique light guiding properties and are attractive for biochemical sensing due to their capability to use cladding as a microfluidic channel [40]. In such a case, the evanescent wave will directly interact with the liquid passing though the holes, enabling potentially high sensitivity by using a small volume of solution while preserving the fiber integrity and structure.

## 3. Metrological Parameters for Assessing Sensor Performance

This section defines and describes the metrological parameters most used in sensing, and is especially focused on those commonly related to OFGs. This section is divided into three subsections: parameters of generic interest attributable to all kind of sensors (Section 3.1), parameters mostly related to refractometers (Section 3.2) and to biosensors (Section 3.3), and some important parameters more specific to grating-based sensors (Section 3.4). However, it is worthwhile pointing out that the entire discussion can also be extended to any resonance-based sensor, such as Fabry-Pérot resonant cavities, interferometers (both Mach-Zehnder and Michelson), whispering gallery mode (WGM) resonators, SPR-based and, recently, lossy mode resonance-based sensors.

### 3.1. Parameters of Generic Interest

There are a number of parameters of generic interest that can be associated with every kind of sensor. These deserve to be mentioned, and are analyzed in the following subsections on uncertainty, accuracy, precision, stability, drift, repeatability, reproducibility and response time. In fact, with the ultimate goal of implementing a commercial device, most of these parameters should be stated in the sensor datasheet.

#### 3.1.1. Uncertainty

In metrology, any measurement should be presented along with its uncertainty (or margin of error). Uncertainty provides a range of values that are most likely close to the “true value” of a given measurement, and is reported in a graph by means of the error bar or denoted by the classical notation measured value ± uncertainty.

Normally, the uncertainty of a measurement is attained by repeating the measurement enough times to get a good estimate of the standard deviation of the measurement values. In this case, any single value has an uncertainty equal to the standard deviation obtained, σ. Nevertheless, if the values are averaged, the mean value of the measurement will then have a much smaller uncertainty, one that is equal to the standard error of the mean value, which is the standard deviation divided by the square root of the number of measurements, σ/√n. However, by following this procedure, systematic errors are overlooked. When the uncertainty is reported by means of the standard error of the measurement, the measured value will fall within the achieved uncertainty range for about 68.3% of the time. By considering a normal distribution of the errors, the standard errors are easily converted to 68.3% (“one sigma”), 95.4% (“two sigma”), or 99.7% (“three sigma”) confidence intervals.

Broadly speaking, the uncertainty depends on both the accuracy and the precision of the sensor. The lower the accuracy and precision of the sensor, the larger the measurement uncertainty.

#### 3.1.2. Accuracy and Precision

Accuracy and precision are two other parameters that are often confused. Accuracy is the deviation between the mean value achievable from a series of measurements (i.e., the data set) and the true value of the measurand assumed as the reference value. Therefore, accuracy represents how close the measurement is to the true value. On the other hand, precision is the level of dispersion of the data set (i.e., the variance, σ^2^) with respect to its mean value. Therefore, precision represents how every single measurement agrees with the other. In general, a measurement can be accurate but not precise, precise but not accurate, accurate and precise, or neither accurate nor precise.

#### 3.1.3. Sensor Drift and Fluctuations

**Figure 6:**
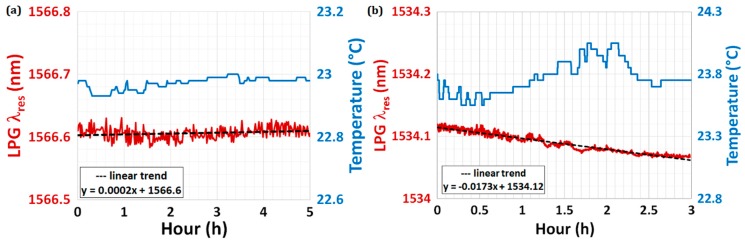
(**a**) Long-term stability test performed using a standard LPG placed inside a thermo-stabilized microfluidic system at a set temperature of 23 °C and (**b**) another LPG laid in an open cell under controlled lab conditions. The blue curves show the thermal fluctuations during the experiment. The red curves account for the time evolution of λ_res_ with the dashed black lines detailing the sensor drift.

The drift and fluctuations of a sensor represent two important parameters, which are often overlooked but deserve more attention since they determine the degree of stability of a sensor. It is clear that the first thing to be considered is the stabilization of the environmental parameters, such as temperature, humidity, possible induced strain, etc. After that, a stability test can be performed by measuring the time evolution of the λ_res_ under a fixed experimental condition (for instance, at 20 °C and in air). As far as the drift is concerned, this can be evaluated from the stability test if the sensing system shows a clear trend during the measurement time. Figure 6 shows an example of a long-term stability test performed using two similar LPGs in two different environmental conditions: the device was placed inside a thermo-stabilized microfluidic system at a set temperature of 23 °C (Figure 6a), whereas the device was laid in an open cell under controlled lab conditions (Figure 6b). In the first case, the drift on λ_res_ (red curve) was 0.2 pm·h^−1^ (practically stable), with thermal fluctuations of ±0.03 °C (blue curve); in the second case, instead, the drift on λ_res_ was 17.3 pm·h^−1^, with thermal fluctuations of ±0.5 °C (one order of magnitude greater).

#### 3.1.4. Repeatability

Repeatability is the level of agreement between a series of measurements of the same measurand, in which every single measurement has been performed with the same sensor under the same experimental conditions. Therefore, the repeatability reflects both the long-term stability of the experimental setup and the sensor lifetime. The error of repeatability influences the uncertainty and represents the lower bound limit of the best precision achievable from the measurements. This parameter is usually expressed in percentages, depending on the unit of measurement used.

#### 3.1.5. Reproducibility

Reproducibility is the level of agreement between a series of measurements of the same measurand, where every single measurement has been performed with the use of different sensors under the same experimental conditions. Therefore, reproducibility reflects the capability of manufacturing and implementing sensors with the same specifications. The error of reproducibility also influences the uncertainty, and represents the maximum deviation measured during a measurement campaign. Reproducibility is usually expressed in percentages, depending on the unit of measurement used.

#### 3.1.6. Response Time

Particular interest is given to the response time of the sensor, which assumes a crucial role in real-time applications. For a generic sensor, the response time is defined as the time required to go from the initial value to a certain percentage of the final value. In the case of the exponential behavior of the sensor response, the response time is usually referred to as the time constant and equals to 1/*e*. Sometimes, especially in chemistry, it is defined as the time required to go from 10% up to 90% of the total variation caused by the change of the measurand of interest [41], also called rise time. The value of the response time can be very different, depending on the sensor.

Speaking of a biosensor, this parameter can be described as the time necessary to reach the equilibrium condition (or plateau) during a binding interaction between the immobilized capture and the analyte under investigation. As a general consideration, the response time depends on a first fast phenomenon, i.e., the kinetics, and on a second slow phenomenon, i.e., the diffusion within the sample that is longer for lower concentrations of analyte [42]. When a coating material (i.e., porous materials, polymers, thin films) is deposited along the sensing region, the response time usually increases due both to the electrostatic interactions between the coating and charged or polar site of the analyte, and to the granularity and homogeneity of the coating [43]. Moreover, the response time of a biosensor can also be influenced by the matrix in which the analyte is spiked: the higher the matrix complexity (more real matrix such as serum, plasma, blood), the longer the response time, since a lower mobility of the analyte is expected [28]. In order to overcome this, the approach involving the measurement of the initial binding rate of the interaction was proposed in the past [44] and recently confirmed [28] as an effective method for determining the analyte concentration.

### 3.2. Parameters Specifically Related to Volume RI Sensing

In this first section, our attention will be focused on the parameters specifically related to devices based on volume RI sensing (see Section 2, such as RI sensitivity and resolution. For each parameter, a definition and a description are provided along with some illustrative figures in order to better highlight the concept.

#### 3.2.1. Response Curve and Refractive Index Sensitivity

**Figure 7:**
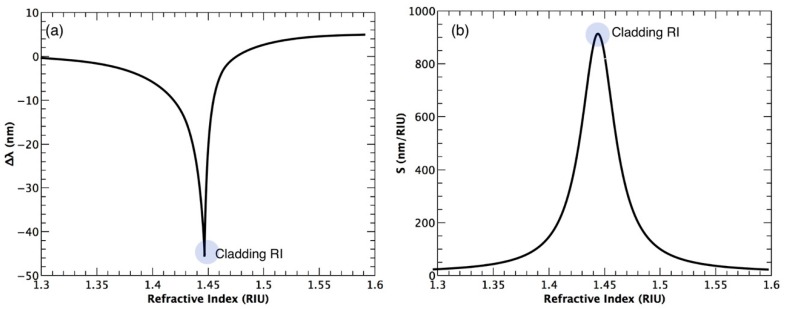
(**a**) A typical response curve of a bare LPG to the SRI in the 1.30–1.60 range (in the case of an EFBG or a TFBG, only the response signal changes) with its corresponding sensitivity; (**b**) A non-linear trend is observed with an increasing sensitivity as the SRI approaches n_clad_. After n_clad_, the sensitivity is considerably reduced again.

The response of an OFG sensor to the SRI, is assessed by monitoring the resonance wavelength (λ_res_) as the SRI is changed. For each SRI evaluated, the value of λ_res_ is averaged within a certain time slot (or within a certain number of measurements), resulting in an experimental point (mean value) and its corresponding standard deviation. Thus, the response curve is built by the set of experimental points with the corresponding error bar (standard deviation). The sensitivity (S) of the sensor to the SRI is given by the derivative of the response curve (∂λ_res_/∂n_sur_), and is expressed in nm·RIU^−1^. It is worth noting that, as also stated in Section 2, the λ_res_ shift of an OFG with the SRI increases (in modulus) in a non-linear trend with the increment of SRI as it becomes closer and closer to n_clad_; after that value, the sensitivity is reduced as it becomes farther and farther away. Figure 7a shows a typical response curve of an LPG and its corresponding sensitivity (Figure 7b) as a function of the SRI. The light grey circles in Figure 7 highlight the region corresponding to the maximum wavelength shift, thus the highest RI sensitivity, when n_sur_ ~ n_cl_. Due to the non-linear behavior, the sensitivity is usually evaluated for a certain index range where a linear approximation can be made. It is also clear that the sensor will be highly sensitive for indices close to n_clad_ and scarcely sensitive for the lower RI range.

#### 3.2.2. Resolution

While calculation of the sensitivity is quite clear-cut, the evaluation of the resolution (R) is more complicated and is often incorrect. It is worth pointing out that R does not depend only on the instrumentation used to measure the resonance wavelength shift (e.g., OSA spectral resolution), but also on the noise sources (e.g., amplitude noise, spectral noise, temperature noise, etc.) and on the method used to process the experimental data. The latter normally involves a fitting procedure, for instance with a Lorentzian or Gaussian function, in order to monitor the wavelength shift of the resonance minimum reliably and effectively.

R can be defined as the smallest variation of the SRI that a sensing device is able to resolve: in other words, the lowest change in the SRI that produces a measurable change in λ_res_. R is proportional to the sensor volume sensitivity (S). As mentioned above, it depends on all the aspects influencing the signal stability that can be experimentally obtained through the standard deviation (σ) of the measurements, with σ affected by all the noise sources, including the algorithm used to calculate the resonance wavelength, repeatability, and also the performance of the interrogation technique. Therefore, the resolution can be calculated trough the expression:

(4) $$R=\frac{p\sigma }{S}$$

where σ is calculated by considering a set of measurements in repeatable and reproducible conditions (thus including all the individual sources of noise) and *p* represents the confidence interval considered as discussed in Section 3.1.1. Most of the time, R is simply evaluated by dividing the instrument spectral resolution by S. In our opinion, this cannot be called resolution, but maximum theoretical resolution (MTR). However, this last issue will be analyzed in detail in Section 6.

In addition, it should be clarified that if R is expressed as RIU, it refers to a refractometer, whereas if R is expressed as nm or pm, it refers to a generic wavelength-based sensing system. Moreover, another important concept to be highlighted, one which is often disregarded, is the fact that R could not be the same throughout the entire sensor working range because of the non-linear behavior of S (Figure 7). This issue, which was clearly brought out in ref. [1], means that it should be considered a minimum and maximum resolution, as occurs for accuracy.

### 3.3. Parameters Specifically Related to Surface RI Sensing

In this section, differently from Section 3.2, our attention will be focused on the parameters specifically related to devices based on surface RI sensing (see Section 2), such as limit of detection, specificity or selectivity and regenerability or reusability. For each parameter, a definition and a description are provided, along with some illustrative figures to highlight the concept better.

#### 3.3.1. Sensorgram and Calibration Curve

**Figure 8:**
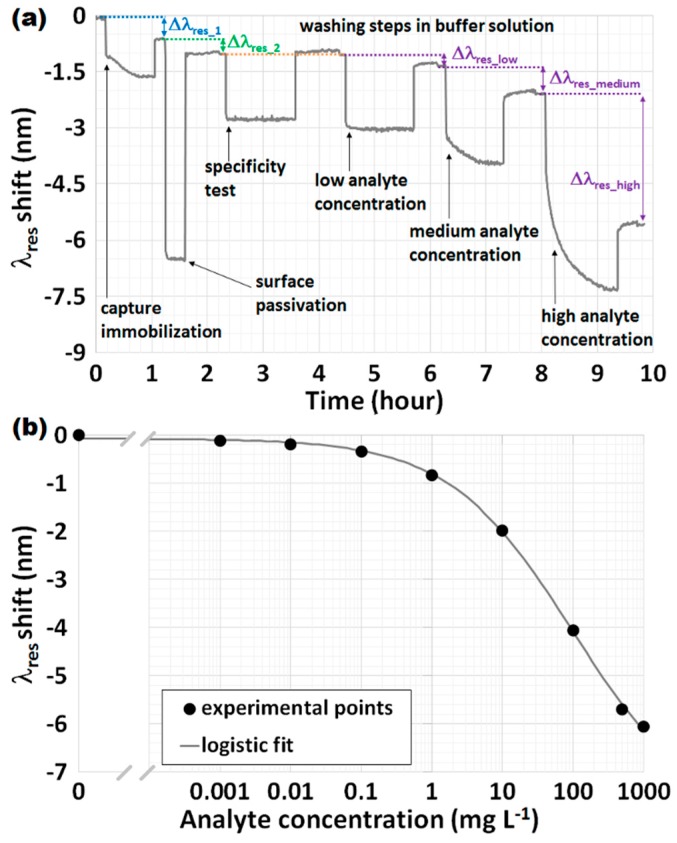
(**a**) An example of a sensorgram reporting the most significant steps of a bioassay highlighted with arrows and the most important wavelength shifts, Δλ_res_, taken after the washing; (**b**) An example of a calibration curve when considering an LPG-based biosensor with the analyte spiked in a serum matrix. The black circles are the experimental points at increasing concentrations (expressed in log scale), whereas the grey line represents the sigmoidal fit of the data using the Logistic function.

A sensorgram is a graph that represents the time evolution of the signal. More specifically, in the case of OFG-based biosensors, the sensorgram shows the evolution of the resonance wavelengths λ_res_ as a function of the time. An example is given in Figure 8a. Clearly, the value of λ_res_ was previously obtained from raw data (i.e., the spectrum) by means of data processing that involves a fitting procedure or other mathematical approaches used to calculate the minimum of the signal. However, it is possible to extrapolate from the sensorgram a series of other important and crucial parameters for a biosensor, such as response time and calibration curve. Figure 8a details the typical steps of a bioassay, which are indicated with arrows, starting from the immobilization of the capture on the sensor surface, followed by the surface passivation to block free remaining functional groups, so as to avoid any non-specific adsorption, and by the specificity test (this will be discussed later in detail, see Section 3.3.3), up to the assay completion carried out by flowing increasing concentrations of the analyte under investigation. After each step, a washing step in a buffer solution, usually a solution of PBS, is performed in order to measure the shift of λ_res_ which is due only to a surface modification. Figure 8a also accounts for some exemplificative shifts of λ_res_, Δλ_res_, taken in PBS after the respective washing when the flow is stopped. Clearly, it a shift is expected after the capture immobilization (blue), surface passivation (green) and analyte interaction (violet), the latter of which increases according to its concentration.

For a biosensor, the calibration curve is a graph that accounts for the signal change with respect to the interacting analyte. More specifically, in the case of OFG, a calibration curve shows the change in the resonance wavelengths λ_res_ as a function of the concentration of the analyte under investigation, which can be expressed in linear or logarithmic scale. The signal can be expressed as the absolute value of λ_res_ or the shift of λ_res_ scaled to the reference concentration that is the concentration at zero analyte, often called blank measurement. An example is given in Figure 8b, where an LPG and the analyte spiked in a serum matrix are considered. The black circles are the experimental points, whereas the grey line represents the sigmoidal fit of the data using the Logistic function, a well-accepted mathematical model for describing the sigmoidal behavior of a biosensor [21,28]. The correlation coefficient (*R*^2^) is greater than 0.999, thus testifying the agreement between the experimental points and the fitting curves.

#### 3.3.2. Limit of Detection

The limit of detection (LOD) represents one of the most important and crucial performance parameter for any biosensor, and should be expressed in terms of a concentration value (C) of the analyte under investigation, such as g·L^−1^ (usually in μg·L^−1^ or ng·L^−1^ considering the molecular weight of the involved analytes) or molarity (M). Given its importance, it is not so difficult to find very different expressions to evaluate the LOD. Moreover, it is also quite common to find articles in which the sensor was tested in a certain concentration range, whereas the claimed LOD is below (even orders of magnitude) the lowest measured concentration value. Since most technical discussions and analytical considerations concerning LOD have already been made in ref. [5], this paragraph reports the different ways in which to evaluate LOD.

In the first approach, the determination of LOD, recommended also by ACS [6], is based on the use of the standard deviations at low concentration. The first step involves repeated measurements (from *k* = 7 to *k* = 10 times) of the blank sample (i.e., the one that does not contain the analyte under investigation) and then the calculation of the mean value, $\bar{y}$_blank_. Afterwards, samples containing the target analyte should be prepared at a concentration of roughly 1–5 times higher than the suspected LOD; *k* measurements are then carried out, and the mean value and the related standard deviation σ_y_ are achieved. The signal at LOD can be calculated as follows:

(5) $$y_{\mathrm{LOD}}={\bar{y}}_{\mathrm{blank}}+\;t_{\alpha ,k-1}\sigma_{y}$$

where $t_{\alpha ,k-1}$ is the α-quantile of t-Student function with *k* − 1 degrees of freedom where (1 − α) defines the confidence interval. All details can be found in ref. [5]. For the sake of simplicity, *t* = 3 is usually assumed in Equation (5), which means a minimum of 16 samples (8 blanks and 8 low concentration samples) have been analyzed within a confidence interval of 99%.

In the second approach, determination of the LOD is based on the use of the calibration curve of the biosensor and on the IUPAC recommendation that states the following: LOD can be obtained from the biosensor calibration curve by considering the standard deviation of the blank measurement three times (3σ_blank_). In the case of a low number of concentrations close to the suspected LOD with a linear response, this approach is quite similar to the first one. On the other hand, if a full calibration curve of the biosensor was obtained, as shown in Figure 8b, a second modified approach is proposed that is a mixture of the two previous ones. LOD can be obtained from the calibration curve through the inverse of the fitting function f^−1^ used (e.g., a sigmoidal curve) evaluated in a well-defined point: the mean value of the blank sample plus three times the maximum standard deviation obtained among all the experimental points (3σ_max_). This can be translated into the following equation:

(6) $$x_{\mathrm{LOD}}=f^{-1}\left({\bar{y}}_{\mathrm{blank}}+3\sigma_{\max}\right)$$

Although the proposed method is different from the recommendations of the standardization organizations, it could represent a valuable option for evaluating LOD within a certain degree of reliability and repeatability (i.e., low value of *k* measurements, *k* < 3).

In the third (and final) approach, the determination of LOD is based on the sensor resolution and on the surface sensitivity (*S*_surface_) [2,4,19], which is the RI change (Δ*n*) divided by the surface density concentration (ρ_max_) of the target analyte, as expressed in the following equation:

(7) $$\mathrm{LOD}=\frac{R}{S_{\mathrm{surface}}}=\frac{R}{\Delta n/\rho_{\max}}$$

In ref. [2], instead of the RI change, the wavelength shift (Δλ) is reported, but it is not correct in view of the dimensional check. In addition, in using this last approach, some authors use the instrumental resolution and the slope of the calibration curve. This would mean that all the measurements fall mostly on the calibration curve, and the standard deviation of the experimental points is lower than the instrumental resolution. However, it seems that the best way to determine LOD would be the first or the second one, even if the third approach is quite common for SPR-based biosensors [42].

**Figure 9:**
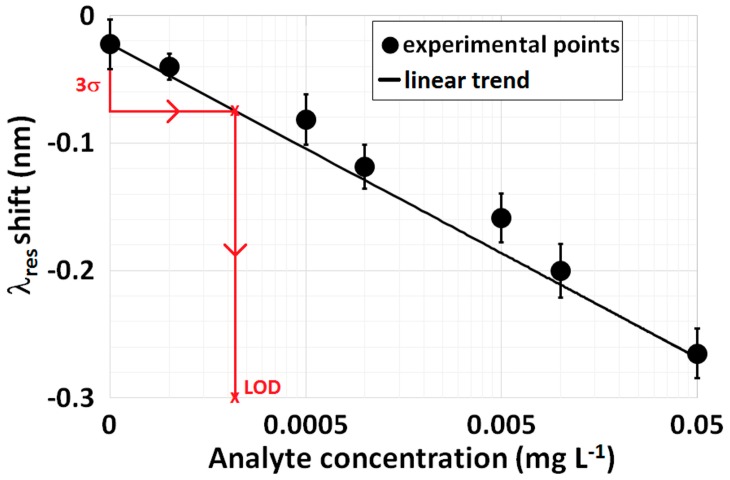
An example of a calibration curve at low concentrations by considering an LPG-based biosensor with the analyte spiked in a serum matrix. The error bars are the result of repeated measurements in a single concentration. The black line provides the linear fit of the data, whereas the red lines make it possible to evaluate the LOD according to the second approach detailed above.

Figure 9 provides another example of a calibration curve at low concentrations achieved with an LPG-based biosensor with the analyte spiked in a serum matrix. The error bars were obtained as a result of repeated measurements at a single concentration, whereas the black lines provide the linear fit of the data. By using the second approach and considering ${\bar{y}}_{\mathrm{blank}}$ = 0.025 nm, the red lines shown in Figure 9 make it possible to evaluate LOD with the following result: 0.23 μg·L^−1^ (σ_blank_ = 0.0168 nm). By using, instead, the second modified approach, the resulting LOD is 0.38 μg·L^−1^ (σ_max_ = 0.0201 nm).

As another example, by looking at Figure 8b and considering the second and second modified Equation (6) definitions of LOD, the following results were achieved by means of repeated tests: 5 μg·L^−1^ (σ_blank_ = 0.016 nm) and 7 μg·L^−1^ (σ_max_ = 0.0189 nm), respectively. Given the more conservative nature of the second modified approach, the LODs were slightly higher than the previous ones. Clearly, the corresponding values of LOD can also be expressed in molarity by using the following equation and knowing the molecular weight (MW) of the analyte under investigation:

(8) $$M\;\left[\mathrm{mol}/L\right]=\frac{C\;\left[g/L\right]}{\mathrm{MW}\;\left[g/\mathrm{mol}\right]}$$

Therefore, Equation (8) makes it possible to report LOD at a concentration value or at a molarity value. As described in refs [23,34], LOD can also be expressed in g·mm^−2^ (usually in pg mm^−2^ by considering the molecular weight of the analytes involved), i.e., in terms of the surface density concentration of the analyte at saturation, as described in the third approach. This approach is typical of SPR-based optical platforms [42].

It is worth pointing out that LOD is different, however, and must not be confused with the MDC of the ISO recommendation [3], which provides the minimum value of the analyte concentration that the biosensor is able to detect reliably. The calculation of this parameter does not imply the utilization of the full calibration curve of the biosensor, but just the use of a few experimental points plus the reference point at 0 analyte concentration (i.e., the blank measurement).

#### 3.3.3. Specificity (or Selectivity)

Specificity (or selectivity) is surely an important parameter for any sensor, but it assumes a key relevance for a chemical and/or biochemical sensor. It represents the sensor’s ability to be sensitive to a specific or a selective measurand, instead of all the other interfering measurands, also called cross-sensitivity. In fact, in practical applications, it is hard or almost impossible to find a sensor that is sensitive to only one measurand. It is worth pointing out that the term “selectivity” is more appropriate for chemical sensors, such as gas sensors, electronic noses and tongues, whereas the term “specificity” is more related to biosensors.

Since all the mathematics and analysis of this parameter have already been carried out [1], this work will focus on a discussion of this parameter as applied to a refractometer and to a biosensor. In the former case, it is well known that an OFG-based sensor is sensitive to a series of measurands, which depend on the kind of OFG [for example strain (axial deformation), temperature, and SRI as the most important]. As already stated, if it is desired to develop an OFG-based refractometer, the effect of cross-sensitivities (strain and temperature) must be taken into account. These works [44,45,46,47,48] are just some of the examples reported in the literature in which some sensor modifications or hybrid sensing configurations were implemented in order to overcome this issue. In this way, the sensor is able to be sensitive precisely and accurately to the SRI changes alone, by getting rid of its intrinsic cross-sensitivities. It should be noted that, in the case of physical sensors, the Community usually speaks about cross-sensitivity, rather than selectivity or specificity.

In the latter case, specificity represents the biosensor’s ability to be sensitive to a specific target analyte. If the measurement is carried out by spiking the analyte in a solution containing just the analyte under investigation (i.e., buffer solution), the biosensor specificity can be tested by injecting a solution containing analytes different from the one under investigation, also known as negative control. On the other hand, if the measurement is performed by spiking the analyte into a more real and complex solution that contains not only the analyte under investigation, but also other analytes (i.e., serum, plasma, blood matrixes in increasing order of complexity), the biosensor specificity can easily be tested by injecting this solution free of the target analyte and measuring the biosensor response. Broadly speaking, the biosensor specificity depends on the affinity of the analyte/bioreceptor pair, the functionalization method, the implemented assay protocol, and the chemistry used in general [7,21].

#### 3.3.4. Regeneration (or Reusability)

Another parameter in biosensing inevitably features regeneration or sensor reusability, thus creating the possibility for real applications. In fact, the use of integrated systems should allow the implementation of an assay protocol that includes a regeneration step in order to obtain the specific biolayer available for a new interaction in consecutive assay cycles, instead of a single usage. Several examples of this kind of regeneration can be found in this recent review on the subject [43]. The other kind involves the restoration of the entire sensing layer, thus removing not only the bound analyte but also the immobilized bioreceptor and the functional layer. This second kind of regeneration can be performed when repeatability and reproducibility have to be evaluated. Given the time-consuming procedure and the requisite of strong treatments often carried out outside the microfluidic system, such as electrochemical cleaning, UV/ozone exposure, ammonia-hydrogen peroxide mixture solution stripping, the first kind of regeneration is the most common one used in biosensing technological platforms.

**Figure 10:**
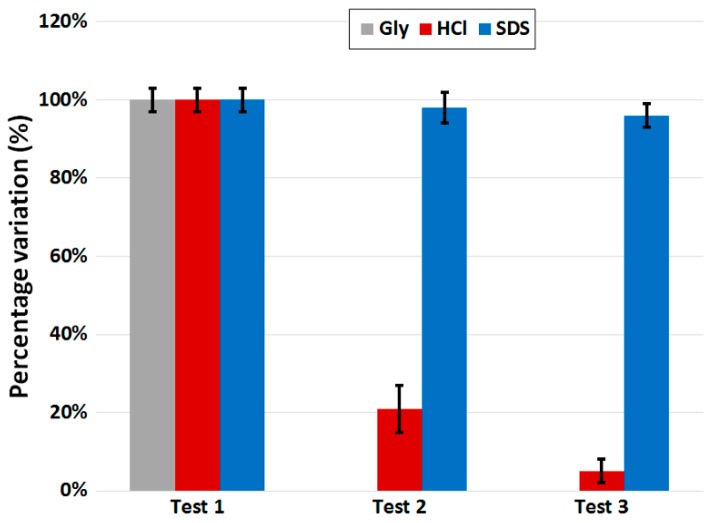
An example of a regeneration test performed and repeated 3 times with the same LPG-based biosensor (with the same anti-IgG/IgG pair) at the analyte concentration of 1 mg·L^−1^ under the same experimental conditions and using three different regeneration solutions: Gly (grey), HCl (red) and SDS (blue). The error bars are the result of multiple measurements at a single concentration. For each sensor, the first value is set to 100% in order to clearly evaluate the amount of signal loss for each cycle due to regeneration.

The first step of regeneration involves the choice of the best regeneration solution as a function of the analyte/bioreceptor pair used [43]. In fact, the same regeneration solution could work expediently for just a specific analyte/bioreceptor pair, and not for any other. Afterwards, in order to evaluate the effect of regeneration, a standard procedure includes the repetition (at least three times) of the measurement of the same analyte concentration after the respective cycle of regeneration. An example is shown in Figure 10, in which the same LPG-based biosensor with the same analyte/bioreceptor pair (i.e., anti-IgG/IgG) was tested three times at the analyte concentration of 1 mg·L^−1^ (~7 nM) under the same experimental conditions and using different regeneration solutions, three of the most common in the literature: glycine (Gly) in grey, hydrochloric acid (HCl) in red and sodium dodecyl sulfate (SDS) in blue. It should be noted that, for each sensor, the first value is set to 100% in order to clearly evaluate the amount of signal loss for each cycle due to the regeneration process. A loss below 5% after the third cycle of regeneration can be considered a good result. Moreover, it is clear from Figure 10 that the best regeneration solution for the analyte/bioreceptor pair used was SDS, whereas Gly seemed ineffective (this is why the graph shows 0% for the second and third tests) and HCl removed almost all the biolayer.

### 3.4. Parameters More Specific to Grating-Based Sensors

#### 3.4.1. Peak Visibility and Bandwidth

The Δλ_res_ or FWHM bandwidth of the resonance peak plays an important role in the performance of the OFG sensors. Since the measurement is based on the determination of the λ_res_, in the resolution estimation it is apparent that the narrower the bandwidth, the more accurate is the detection of the λ_res_. This comes from the finite number of possibilities within the bandwidth of the resonance. Therefore, the resolution of the sensor will be inversely proportional to Δλ_res_. In fact, if the performances of FBG- and LPG-based refractometers are compared, the big difference between the Δλ_res_ (with a much larger value in the case of LPGs) could imply that an FBG with a much lower RI sensitivity than an LPG exhibits a better resolution. On the other hand, this aspect has impact even when the performance of an LPG is compared at different SRI ranges. It is well known that the sensitivity is much higher close to n_clad_, but also that the resonance becomes sharper and considerably wider.

Another parameter that can strongly influence the performance of a resonance-based device, especially the resolution and LOD, is the shape and symmetry (or simply the peak visibility) of the attenuation band considered. In fact, especially when an overlay is deposited along the region containing an OFG, both the FWHM tends to increase and the peak visibility tends to worsen with a decrease in the peak symmetry. Although this depends on the quality and methodology of the deposition, it represents an aspect that should not be disregarded and, indeed, should be taken into account.

#### 3.4.2. Influence of the λ_res_ Determination on RI Sensitivity and Resolution

Another important parameter that has considerable impact on sensor performance is the interrogation technique, which is related to the way in which the resonance wavelength is determined. A common practice is based in a simple peak-finding algorithm, which locates the maxima or minima of the spectrum. However, the performance of this approach, although very simple, leads to modest results, especially in cases of low signal-to-noise ratio. In alternative, there are other algorithms with increased complexity that first apply a filter to remove high frequency noise and then use a Lorentzian (or Gaussian) fitting or a centroid calculation to evaluate the λ_res_. Both of these show very good accuracy and precision. On the other hand, there are other techniques based on intensity measurements, in which the laser emission is modulated in intensity as a function of the wavelength deviation. This method requires a self-referring scheme to compensate for power fluctuations in the system. Although its performance is good, it is limited by a short range of variation of the SRI because of the shape and bandwidth of the grating spectrum [45].

## 4. Refractometers

This section summarizes the results achieved in the literature using both FBGs (Section 4.1) and LPGs (Section 4.2) as optical refractometers. This means that the sensor measures the shift of the λ_res_ induced by volume RI changes (see Figure 5a). In this case, the device consists of a bare optical fiber or of an optical fiber coated with an overlay, which is used only to enhance the RI sensitivity and does not act as a selective sensing layer able to capture a biological species or to change its characteristics (e.g., the overlay RI and thickness) because of a chemical/biochemical interaction, as in the case of surface RI changes (see Figure 5b).

The interfering cross-sensitivities (temperature, strain and curvature) represent a crucial issue related to OFG-based refractometers because they influence the detection capabilities and performances of these devices. In fact, one of the major constraints of grating-based sensors to effectively achieve high resolution RI sensing is the temperature cross-sensitivity. It affects, to a greater or lesser degree, all optical fiber sensors, and arises from the intrinsic dependence of the silica matrix to temperature, through the thermo-optic and thermal expansion coefficients. Thereby, changes in temperature will induce changes both in the effective RI and in the effective length of the grating, thus varying the optical signal [46]. These changes may be higher or lower, depending on the fiber composition and the characteristics of the grating. Typically, the thermo-optic coefficient of silica is 8.0 × 10^−6^ K^−1^, whereas its thermal expansion coefficient is 0.55 × 10^−6^ K^−1^. These values are mostly related to silica fibers, and may change slightly, depending on the fiber composition. In fact, the dopants (for instance, germanium, boron, etc.) used for improving fiber photosensitivity, an essential aspect for their fabrication, may introduce stronger temperature dependences [47]. At present, in most practical applications, authors deal with temperature dependence by working with temperature-stabilized systems. Nevertheless, even when the temperature is stabilized to ±0.1 °C, such fluctuations can still be significant in the face of a need to measure variations of RI down to 10^−5^ RIU or even lower.

Besides temperature, OFG sensing devices based on the evanescent field are also sensitive to strain and curvature. Usually, the sensing portion of the fiber is well fixed inside a chamber or an ad-hoc developed packaging in order to keep it straight or under a little tension. However, a small gap in the anchoring points can generate a drift on this tension over time. Moreover, a variation in the environmental temperature can lead to changes in the applied tension due to the elasto-optic coefficient of the materials constituting the chamber. These small changes will affect the optical response of the sensor, implying an important limitation in the SRI resolution of the sensing device.

Cross-sensitive issues are often solved by utilizing more elaborated schemes, for instance, by using cascaded gratings [44,48] to measure and compensate the changes of the interfering physical parameters or by using a differential arrangement [46]. In any case, the use of a mechano- and thermo-stabilized microfluidic system greatly helps to reduce this problem to a minimum [48].

### 4.1. Fiber Bragg Gratings

Asseh et al. [10] presented in 1998 the first demonstration of an FBG-based refractometer: an FBG written in a SMF fiber was etched, until the diameter of the fiber was reduced to 11 μm. The sensitivity was 1 nm·RIU^−1^ for refractive indices close to 1.33 RIU and the maximum theoretical resolution was 2.5 × 10^−4^ RIU. Major enhancements in sensitivity were presented by Chryssis et al. [49] by exposing the core of the fiber by means of etching processes. A maximum sensitivity of 1394 nm·RIU^−1^ was achieved as the SRI approaches the core index, when the fiber diameter was reduced to 3.4 μm. These EFBG configurations are undoubtedly interesting, and can reach good performance levels to the detriment of sensor robustness, handling and practical applicability. Liu et al. [12] presented a FBG written in a 1.8 μm diameter microfiber by using focused ion beam milling to directly etch periodical structures on the surface. The device showed a maximum sensitivity of 660 nm·RIU^−1^ for refractive index values near 1.38 RIU.

Dealing with refractometers based on standard FBGs, Schroeder et al. [11] presented two in-line FBGs written on a SMF, where one of them was side-polished to become sensitive to the SRI, whereas the second one was used for temperature compensation. The effect of HRI overlays and the operation at longer wavelengths was also studied so as to enhance its RI sensitivity. The maximum sensitivity for an SRI close to 1.45 RIU was found to be 300 nm·RIU^−1^ and the maximum theoretical resolution was 2 × 10^−6^ RIU. A simpler and more effective solution for temperature compensation was reported by Iadicicco et al. [50]: a single grating was half-etched with the result of the split of the original attenuation band into two peaks, with one of them sensitive to the SRI changes and the other one sensitive only to temperature variations. Liang et al. [51] reported an RI fiber sensor based on a Fabry-Pérot interferometer formed by two identical FBGs and separated by a portion of fiber with a radius of 1.5 μm. The device showed a sensitivity of 71.2 nm·RIU^−1^ near 1.33 RIU and a resolution (by considering the fluctuations on the resonance wavelength) of 1.4 × 10^−5^ RIU.

Another effective, highly investigated and feasible option for accurate refractometry is provided by TFBGs. Laffont et al. [13] presented a TFBG with a tilting angle of 16° for RI sensing. The SRI measurement was retrieved by tracking the normalized envelope of the cladding mode resonances spectrum. This parameter was shown to be insensitive to temperature changes, and made available a linear operation range from 1.32 RIU to 1.42 RIU, with a resolution of 10^−4^ RIU. Later, Chan et al. [15] proposed a relative measurement of RI based on the separation distance between a specific cladding mode, which is dependent on the RI and temperature changes, and the core mode, which is refractive index independent. A 4° TFBG was used, and a RI sensitivity of 10 nm·RIU^−1^ was obtained in the range (of RI) between 1.38 RIU and 1.43 RIU, enabling a maximum theoretical resolution of 10^−4^ RIU. Moreover, Zhou et al. [14] reported on an 81° super-tilted FBG that was capable of attaining a sensitivity of 340 nm·RIU^−1^ close to 1.33 RIU. Recently, an HRI coated TFBG was presented by Renoirt et al. [29]. The device consists of a 6° TFBG coated with a 200 nm thick film of zinc oxide. The results showed a sensitivity of 210 nm·RIU^−1^ and a maximum theoretical resolution of 10^−5^ RIU for refractive indices close to 1.33 RIU. The same research group demonstrated a 10° TFBG coated with an oriented monolayer of silver nanowires as a SPR sensor [52]. A sensitivity of 650 nm·RIU^−1^ was achieved for indices close to water environments.

Dealing with refractometers based on FBGs written in a suspended core MOF, Huy et al. reported both a standard FBG [39] (three holes MOF) and a TFBG [38] (six holes MOF). In this situation, the liquid filling the holes affects the core mode of the FBG. The device, which is based on a standard FBG, presented a sensitivity of 210 nm·RIU^−1^ and a maximum theoretical resolution of 3 × 10^−5^ RIU at 1.33 RIU [39]. In the case of the 6° TFBG [38], a maximum theoretical resolution of 2.3 × 10^−4^ RIU at 1.33 RIU was attained.

### 4.2. Long Period Gratings

For the very first time, in 1996 Bhatia et al. [16] studied the application of LPGs for refractive index sensing. An LPG, with a period of 320 µm written by UV radiation exposure in a standard SMF, exhibited a wavelength shift of 62 nm for a RI change between 1.40 and 1.45 RIU and a maximum theoretical resolution of 7.69 × 10^−5^ RIU in the same RI range.

Subsequently, a comprehensive study regarding the dependence of the cladding mode order with the sensitivity of LPGs was described in detail by Shu et al. [30]. Different gratings were written in boron–germanium co-doped (photosensitive) fiber by means of a UV laser irradiation technique, with grating periods ranging from 34 µm to 700 µm. The best performance was shown by a LPG close to its own TAP, with a grating period of 202 µm that excited the 11-th cladding mode order at around 1550 nm. An RI sensitivity of 1481 nm·RIU^−1^ in the RI range from 1 to1.36 RIU was shown: as far as we know, this is the best RI sensitivity for a bare LPG reported so far for this RI range. This work then opened up the way to novel sensing design approaches with LPGs.

Following this strategy, Pilla et al. [18] recently reported an LPG close to the TAP coated with an HRI overlay. The UV-written LPG with its period of 200 µm was coated with a polystyrene film. The coating thickness was approximately 245 nm. For the 11-th cladding mode order resonance, a RI sensitivity of over 9000 nm·RIU^−1^ close to 1.347 RIU was achieved. Similarly, Smietana et al. [53] presented an LPG near the TAP coated with thin titanium dioxide (TiO_2_) overlays. With a thickness of 70 nm, a RI sensitivity of 6200 nm·RIU^−1^ close to 1.34 RIU was achieved. Very recently, Chiavaioli et al. [28] described an evanescent wave optical fiber sensor based on a TiO_2_–SiO_2_-coated LPG. The thickness of the sol-gel-based titania-silica overlay was approximately 159 nm. For the 7-th cladding mode order resonance, a RI sensitivity exceeding 7000 nm·RIU^−1^ close to 1.333 RIU and a resolution of 4.6 × 10^−6^ RIU were achieved. Therefore, these results have classified thin film coated LPGs as an effective and compelling alternative to other fiber-based technologies for high-performance chemical and biological sensing applications, as will be shown in the following section.

With reference to the fabrication processes, when compared to UV or femtosecond laser radiation techniques, electric-arc induced LPGs are attractive in any case due to their simplicity and flexibility, as well as the lower cost of the fabrication process [54]. Debowska et al. [55] published a work comparing performances between bare LPGs and HRI coated LPGs written by using the electric-arc technique in photosensitive PS1250/1500 fiber, with grating periods of around 190 µm close to the TAP. The devices were coated with a 30-nm-thick silicon nitride overlay. The results showed RI sensitivities of 780 nm·RIU^−1^ and 2146 nm·RIU^−1^ in the RI range from 1.33 to 1.36 RIU for the bare and coated devices, respectively. These represent the highest sensitivities reported for bare and coated LPGs with the use of the electric-arc technique for the specific measuring range.

In 2006 Rindorf et al. [37] presented the first LPG written on a pure silica (LMA10) MOF for refractometric sensing. The LPG with a grating period of 700 µm was written using a CO_2_ laser inscription technique. The device exhibited a sensitivity of roughly 400 nm·RIU^−1^ close to an RI of 1.33 RIU and a resolution of 10^−4^ RIU. Subsequently, He et al. [56] demonstrated an important improvement as regards the sensitivity of LPGs written on a MOF using a CO_2_ laser source. With a grating period of 430 µm, the device showed a sensitivity of 2991 nm·RIU^−1^ and a maximum theoretical resolution of 3.3 × 10^−7^ in the RI range between 1.33 and 1.35 RIU.

Lastly, another interesting and extensively studied configuration is given by LPG-based interferometers. In the LPG-based Mach-Zehnder interferometer realized by Allsop et al. [57], a pair of LPGs (Λ = 270 µm, coupled with the 9-th cladding mode order resonance) at a distance of 10 cm one from the other was used and the LPGs were interrogated by a phase-generated carrier technique. A resolution of the order of 10^−6^ RIU was achieved for a RI range of between 1.37 and 1.40 RIU. Subsequently, Mosquera et al. [58] reported a high finesse Fabry-Pérot fiber cavity that incorporates an intra-cavity LPG, which couples and recovers energy to the fiber cladding after being phase-shifted by the SRI. The cavity consisted of two high-reflectivity (~95%) FBGs separated by a distance of 47.5 mm. The SRI is monitored by means of the resonant frequencies of the Fabry-Pérot interferometer. A resolution of 2.1 × 10^−5^ RIU was achieved for n_sur_ = 1.33 RIU.

Table 1 gathers together the most relevant grating-based optical fiber platforms for refractive index sensing reported up until now, along with the performances achieved in terms of RI sensitivity and resolution as concern. As can be observed and as previously mentioned, most of the papers do not report the resolution or, at least, provide a value that is actually the MTR truly. Very few of them account for the real resolution calculated using Equation (4) [28,51,57].

## 5. Biosensors

This section summarizes the results achieved in the literature using both FBGs (Section 5.1) and LPGs (Section 5.2) as biosensors. This means that, due to a chemical/biochemical interaction, the sensor measures the shift of the λ_res_ induced by surface RI changes, as shown in Figure 5b. In this case, the optical fiber containing the grating is coated with an overlay, which serves not only to enhance the RI sensitivity but also to act as a selective sensing layer capable of capturing a biological species and thus of changing its characteristics (e.g., the average RI and thickness).

Recalling what has been said about interfering cross-sensitivities for refractometers, this also represents an important issue for OFG-based biosensors. In particular, temperature not only influences the RI measurement, but also greatly affects the interaction between two molecules or two biological species. Therefore, the sensor response to a biological assay is expected to be different if the assay is performed at 23 °C, rather than at 30 °C or 37 °C. This means that, due to the fact of having a thermal- and mechanical-controlled microfluidic system in which the OFG-based biosensor can be integrated, it is crucial to attain reliable, effective, accurate and repeatable results. In fact, the main technological components of a biosensors should consist of an optical sensing platform, a microfluidic system, and a biological recognition element (BRE) used specifically to capture the analyte being investigated. Clearly, the choice of a BRE immobilization method (such as covalent binding, hydrophobic interaction, electrostatic interaction, etc.) can play an important role, and depends on the type of functional molecule, on the size of the target analyte, and on the application specifics [8]. Important issues that must be considered in the BRE immobilization and that can heavily influence the biosensor performance are their surface density and their orientation after the immobilization, as well as the maintenance of their functional properties as regards the analyte being tested.

### 5.1. Fiber Bragg Gratings

The earliest demonstration of a biosensor based on FBGs was made by Chryssis et al. [59] in 2005. The sensing head was based on an EFBG, on the fiber surface of which single stranded DNA oligonucleotide probes of 20 bases were immobilized. Hybridization of a complementary target single-strand DNA oligonucleotide was detected. Sun et al. [60] demonstrated a microfiber FBG for the detection of DNA hybridization. The biosensor showed a lowest detectable concentration of 0.5 mM. On the other hand, Candiani et al. [61] studied FBGs written in MOF for the detection of specific DNA target sequences. The inner surface of the MOF was functionalized with a peptide nucleic acid probe, while gold nanoparticles were used in a sandwich assay for signal enhancement.

Maguis et al. [19] studied the recognition and interaction kinetics of a specific antibody-antigen pair based on a TFBG structure by changing the antibody concentration in different configurations for the antigen immobilized on the fiber surface. Bovine serum albumin (BSA) (antigen) and anti-BSA (antibody) were used in this study. Afterwards, Lepinay et al. [62] showed improvements in the response of TFBG-based biosensors by using both gold nanospheres and nanocages. These two types of nanoparticles were used to enhance the light interaction and localization, thus improving the resonance shifts in the fiber transmission spectrum. The system was tested by monitoring the binding of biotin to avidin immobilized on the fiber surface. An impressive LOD of roughly 8 pM was obtained. Super tilted FBGs were also explored for biosensing applications. Luo et al. [63] presented an enzyme-functionalized TFBG biosensor for glucose determination. The fiber surface of an 81° TFBG was modified by glucose oxidase. A sensitivity of 0.298 nm·(mg/mL)^−1^ was obtained in a concentration range of 0.0–3.0 mg·mL^−1^.

A different approach was presented by Shevchenko et al. [64], using a SPR-based TFBG sensor. The sensor surface was functionalized with high specificity synthetic DNA sequences (aptamers). The biosensor showed an ability to detect and quantify various concentrations of thrombin, with a LOD of roughly 23 nM. Later, the same authors proposed the application of the same sensing head (i.e., SPR-based TFBG biosensor) for the real-time monitoring of cellular responses [65]. The cells were stuck to the gold-coated surface that was modified with fibronectin, a cell-adhesive protein. The influence of various chemical stimuli on cells - trypsin, serum and sodium azide-was studied. These stimuli induced the detachment of cells from the sensor surface. Recently, Malachovska et al. [66] demonstrated the feasibility of using SPR-based TFBGs for specific single cell-sensing and for targeting membrane proteins in intact cells. The device is capable of selectively detecting intact epithelial cells as analytes in cell suspensions in the range of 2–5 × 10^6^ cells·mL^−1^.

### 5.2. Long Period Gratings

The first demonstration of a fiber optic device for biomolecule detection using an LPG was presented in 2000 by DeLisa et al. [44]. In it, the LPG was used to detect and monitor antibody-antigen interactions. Goat anti-human immunoglobulin G (IgG) was immobilized on the fiber surface containing the LPG, and the binding of the specific human IgG (antigen) binding was shown in the concentration range of 2–100 μg·mL^−1^. Afterwards, several works reported on studies regarding antibody-antigen interaction using different configurations of LPGs, such as LPG in a bare optical fiber [67], an acoustic-generated LPG [68], TAP LPGs [69], HRI coated LPGs [27,70,71], LPG-based Michelson interferometers [72,73], and MOF LPG [74].

Cooper et al. [75] demonstrated the application of an LPG biosensor based on immunological binding for *Francisella tularensis* detection. The LPG was functionalized with the IgG fraction of antiserum to *F. tularensis*. The presence of the bacteria was detected by means of the binding of specific antigen.

Chiavaioli et al. [76] focused in detail on the study of antibody-antigen (IgG/anti-IgG) interactions by using different types of LPG-based biosensors integrated into a home-made thermo-stabilized flow cell. In the first work, the authors used a standard LPG in which the coupling occurred with the 7th cladding mode, with the fiber surface functionalized by using a methacrylic acid/methacrylate copolymer (Eudragit L100). The device showed a LOD of 500 ng·mL^−1^. At a later date, the same authors presented a ten-fold performance enhancement by using a TAP LPG, in which the coupling occurred with the 12th cladding mode, and showed a LOD of 50 ng·mL^−1^ [77]. Recently, using a titania-silica coated LPG that was optimized to work in the transition region of the 7th cladding mode, the same authors accounted for a further improvement in LOD, reaching values of 13 ng·mL^−1^ and 8 ng·mL^−1^ (roughly, 53 pM) that were achieved in buffer solution and in serum matrix, respectively [28].

Chen et al. [69] demonstrated an LPG close to the TAP for the detection of DNA hybridization. The DNA probe was covalently immobilized on the grating surface and was subsequently hybridized with the complementary target DNA sequence. Recently, the same authors presented an improvement in the immobilization of the DNA probe, one that allowed a detectable oligonucleotide concentration of 4 nM [78]. Other researchers have explored DNA hybridization by using LPG sensors [79,80,81] and LPG in MOF [37].

Carrasquilla et al. [82] presented an LPG-based Michelson interferometer with the immobilization of aptamers along its sensing path (in between the two LPGs), thus making possible to attain a LOD of 400 μM for adenosine triphosphate. Later, Queirós et al. [83] reported an LPG functionalized with aptamers for the detection of *E. Coli* outer proteins with a LOD of 1 nM.

LPGs applied for label-free detection of specific bacteria using physically adsorbed bacteriophages were reported for the first time in 2011 by Smietana et al. [84]. In this case, T4 phages immobilized onto the fiber surface containing the LPG were used as a recognition element for *E. coli* detection. A similar work with an enhancement in the recognition capability was presented by Tripathi et al. [85], and showed reliable detection of *E. coli* concentrations as low as 10^3^ cfu·mL^−1^ (cfu, colony forming units). Furthermore, Brzozowska [86] et al. recently presented an LPG functionalized with endotoxin (*E. coli B lipopolysaccharide*) binding protein (adhesin) of T4 bacteriophage. Using the same chemistry, improved recognition capability in the detection of bacteria endotoxin was obtained by Smietana et al. [53]: in their work, the fiber surface of a TAP LPG is coated with a thin layer of titanium dioxide, thus making the mode transition possible.

Finally, an enzyme-coated LPG was used for the detection of glucose by Deep et al. [87], by means of the immobilization of glucose oxidase on the fiber surface. Baliyan et al. [88], instead, presented a lipase enzyme-coated LPG for the detection of triacylglycerides in human blood. The authors showed the effect of pH on the sensor response, and the best performance, i.e., 0.5 mM, the lowest detectable concentration, was achieved at a temperature of 37 °C and pH of 7.4.

Table 2 contains a collection of some of the most relevant grating-based optical fiber platforms for different biosensing applications reported up to now, for which the performance achieved in terms of detection range and LOD (or, alternatively, the lowest detectable concentration) are reported with great clarity.

## 6. Some Common Mistakes and an Attempt to Bring Uniformity to Performance Evaluation

As regards the refractometers based on an OFG configuration, the most common mistake present in more than 50% of the related literature concerns evaluations of the sensor resolution, by considering in Equation (4) the OSA spectral or power resolution, instead of the standard deviation of measurement such as noise (see Table 1 and Section 4.2). As mentioned in Section 3.2.2 and if we take a good look at Table 1, this cannot be considered the actual resolution of the device; however, at least the MTR is indeed an indication of the sensor performance. In fact, the instrument resolution can represent simply a part of the noise sources. Without a noise test of the entire sensing scheme, it is impossible to evaluate the resolution.

The other and large part of mistakes related to biosensors implemented by OFG configurations concerns the LOD evaluation. In some articles, LOD is reported as a function of (volume) RI changes that truly indicate the sensor resolution. Another really common error in the biosensor literature, and one that is often overlooked, is that LOD cannot be determined with a single measurement close to the foreseeable LOD, because it is impossible to evaluate the σ of the measurement from a single measurement. Moreover, there are articles that contain very small amounts of experimental points, in which the authors estimate the LOD by providing a value well below the lowest concentration tested in the experiment.

In order to provide a performance evaluation of grating-based sensing devices that is as uniform as possible (valid in general also for any resonance-based sensing device) and to allow for a direct comparison with the other already-published sensors, one should carefully consider this itemized list of the most important parameters to be declared for a refractometer and for a biosensor, how to express each of them, and how the impact is of each parameter to identify and to quantify the measurand under investigation. In the case of a refractometer, at least the following should be stated:

- Volume Sensitivity (S) expressed as nm·RIU^−1^ (wavelength interrogation) or as dB·RIU^−1^ (intensity interrogation) and as shown in Section 3.2.1;
- Resolution (R) expressed as RIU and as fully discussed in Section 3.2.2;
- Accuracy expressed as RIU and as described in Section 3.1.2.

It is worth remembering that all the previous parameters can show different values, depending on the working range of the sensor: that is to say the considered range of refractive index, which in turn has to be declared.

In the case of a biosensor, the following points should be stated:

- Response Time, expressed in seconds or in minutes, which can be derived from the sensorgram for each concentration of the target analyte as well as from the binding kinetics;
- Limit of Detection (LOD), expressed in terms of concentration C (g·L^−1^ or M), which can be obtained from the calibration curve, as fully discussed in Section 3.3.2;
- Specificity, which can be tested by using a complex matrix or by performing a negative control during the assay implementation, as discussed in Section 3.3.3;
- Reusability, expressed in terms of percentage, which can be evaluated by performing at least three cycles of regeneration tests with the same sensor, as shown in Section 3.3.4;
- Surface Sensitivity, expressed as the RI change divided by the surface density concentration of the target analyte. This is not mandatory but it could provide additional information, as discussed in Section 3.3.2.

## 7. Conclusions

The results presented confirm that grating-based sensors represent a promising tool for lab-on-fiber technology platforms and that they have the potential to be used as disposable devices for in situ and real-time clinical applications. In view of the increasing number of communities related to optical fiber sensors, especially those based on a resonant attenuation band both in the visible region and in the infrared one, the aim of the proposed work was to provide the Research and Industrial Communities with a tool for a more uniform and direct comparison among the members of this large field by defining and pointing out the most crucial parameters to be used to establish the performance of any sensor. In any case, a summary of the definitions of the most important parameters for refractive index sensing and biosensing and their use in common applications is provided in Appendix A, with the aim of ensuring a direct and enlightening understanding so as to avoid the typical mistakes present in the literature

In addition to all the discussion made on the three most essential performance parameters, i.e., sensitivity, resolution and limit of detection, it should also be taken into account that the shape of the resonant band (or visibility, as it is called in Section 3.4.1), and also its bandwidth play an important role in the extraction method for evaluating the resonance wavelength and thus in its accuracy and precision. This fact dramatically impinges on the sensor resolution (refractometer) as well as on the LOD (biosensor). The effect of a non-symmetric, wrapped and flattened resonance is often underestimated in some of the literature, and certain highly sensitive devices encompassing these characteristics cannot reach equally high performance levels in terms of resolution and LOD. Moreover, both the sensor stability and drift discussed in Section 3.1.3 represent two other parameters that most of the literature in the field often disregards, while they play an important role in any biosensing application involved in the realization of prototypes or industrial devices. Two other parameters that most of the literature in the field overlooks at times are repeatability and reproducibility. If they are related to the sensor manufacturing and the measurements, they are surely crucial for real applications in the direction of a development of finalized devices to be put on the market.

Finally, it is worth noting that if a proof-of-concept device is presented, the performance parameters listed in the previous section can be disregarded; however, if a fully-developed device is described, those parameters should at least be specified. In fact, a declaration of all those parameters should surely make any comparison easier and should help not only reviewers and readers, but also Industry, to select the best-fitted device as a function of the required application. Therefore, it is still true (and even more appropriate and effective) to say, “More information, more knowledge”.

## Figures and Tables

**Table 1:** Performance comparison of the most relevant grating-based optical fiber platforms for refractive index sensing in terms of refractive index sensitivity and resolution.

Type of Sensing Structure	RI Range (RIU)	Sensitivity (nm·RIU^−1^)	Resolution (RIU)	Ref.
EFBG	~1.46	1394	7.2 × 10^−6 †^	[49]
Microfiber FBG	~1.38	660	-	[12]
81° TFBG	~1.33	340	-	[14]
HRI coated TFBG	~1.33	210	10^−5 †^	[29]
SPR-based TFBG	~1.33	650	-	[52]
Fabry-Pérot cavity-based FBG	~1.33	71.2	1.4 × 10^−5^	[51]
FBG in MOF	~1.33	210	3 × 10^−5 †^	[39]
TAP LPG	1.33–1.36	1481	-	[30]
HRI coated TAP LPG	~1.35	9100	-	[18]
HRI coated LPG	~1.33	7075	4.6 × 10^−6^	[28]
LPG in MOF	1.33–1.35	2991	3.3 × 10^−7 †^	[56]
Mach-Zehnder-based LPG	1.37–1.40	-	1.8 × 10^−6^	[57]

^†^ Maximum theoretical resolution (MTR) as defined in Section 3.2.2.

**Table 2:** Performance comparison of the most relevant grating-based optical fiber platforms for different biosensing applications in terms of detection range and LOD.

Type of Sensing Structure	Biosensing Interaction	Detection Range	LOD	Ref.
Microfiber FBG	DNA hybridization	0.5–20 μM	0.5 μM ^†^	[60]
TFBG	BSA/anti-BSA	1–100 mg·L^−1^	0.086 mg·L^−1 ‡^	[19]
TFBG with gold nanospheres and nanocages	Avidin/Biotin	10 pM–100·μM	8 pM	[62]
81° TFBG	Glucose	0–3 g·L^−1^	0.1 g·L^−1 †^	[63]
SPR-based TFBG	Aptamer/Thrombin	0.1–5 μM	22.6 nM	[64]
SPR-based TFBG	Single cell and membrane proteins	0.5−5 × 10^6^ cells·mL^−1^	2 × 10^6^ cells·mL^−1 †^	[66]
LPG	IgG/anti-IgG	2–50 mg·L^−1^	2 mg·L^−1 †^	[44]
TAP LPG	Biotin/Streptavidin	0–100 mg·L^−1^	12.5 mg·L^−1 †^	[68]
LPG	IgG/anti-IgG	0.1–500 mg·L^−1^	0.5 mL^−1^/3.33 nM (buffer)	[75]
TAP LPG	IgG/anti-IgG	0.001–100 mg·L^−1^	70 μg L^−1^/460 pM (serum)	[77]
HRI coated LPG	IgG/anti-IgG	0.001–100 mg·L^−1^	8 μg L^−1^/53 pM (serum)	[28]
TAP LPG	DNA hybridization	0–5 μM	4 nM	[78]
LPG	DNA hybridization	-	62 nM ^§^	[81]
Michelson-based LPG	Aptamer/Adenosine triphosphate	0–2.5 mM	400 μM	[82]
LPG	Aptamer/*E. coli* outer proteins	0.1–30 nM	1 nM	[83]
LPG	Bacteriophages/*E. coli*	0–10^9^ cfu·mL^−1^	10^3^ cfu·mL^−1 †^	[84]
LPG	Glucose/enzymatic	0.1–3 g L^−1^	-	[87]
LPG	Triacylglyceride/enzymatic	0–7 mM	0.5 mM ^†^	[88]

^†^ Lowest detectable concentration as defined in Section 3.3.2. ^‡^ Evaluated from the affinity constant and sensor resolution, as discussed in Section 3.3.2. ^§^ Evaluated from the slope of the fitted calibration curve, as discussed in Section 3.3.2.

## Appendix A

Here, a summary of the definitions of the most important parameters for refractive index sensing and biosensing and their use in common applications is provided with the aim of a direct and enlightening understanding in order to avoid the typical mistakes present in the literature. As stressed in the paper, those parameters have been discussed and analyzed by considering grating-based sensing devices (both fiber Bragg gratings and long period gratings), but their definitions and applications can also be extended to all resonance-based sensors and biosensors thus providing the basis for an easier and more direct performance comparison of a great number of sensors published in the literature up to now.

In the case of *volume RI sensing* (refractometer), the most important parameters to detail are three (at least):

1. RI sensitivity (nm·RIU^−1^), the derivative (the slope) of the response curve, in the particular RI range when the sensor response is not linear. Specifically, this value should be reported around the RI of water with a view to an application as a biosensor.
2. Resolution (RIU), the smallest variation in the surrounding RI that the sensing device is able to resolve or the lowest change in the surrounding RI that produces a measurable change, in the particular RI range when the sensor response is not linear. Specifically, the influence of the noise sources and their contributions should be addressed when the “real” resolution is provided. Otherwise, one should talk about the maximum theoretical resolution attainable from the sensor by considering the instrument resolution. Clearly, in an evaluation of the resolution, certain considerations related to the visibility (i.e., shape and intensity) and bandwidth of the resonance have to come out. Therefore, the resolution is also indirectly related to the figure of merit (Q-factor for instance) of the resonance, which should be detailed.
3. Accuracy (RIU), the deviation between the mean value achievable from a series of measurements (i.e., the data set) and the true value of the measurand assumed as the reference value, in the particular RI range when the sensor response is not linear. Specifically, this parameter guarantees that the device has been tested with repeated measurements, thus providing an idea of repeatability and reproducibility.

In the case of *surface RI sensing* (biosensor), the most important parameters to be detailed are three (at least):

1. Limit of detection (M, g·L^−1^ or g·mm^−2^), three times the standard deviation of the blank measurement from the biosensor calibration curve obtained with repeated measurements (k). Alternatively, another approach has been proposed: three times the maximum standard deviation obtained among all the experimental points of the calibration curve that usually corresponds to the maximum deviation from the fitting function. This latter approach represents a valuable option for evaluating the LOD within a certain degree of reliability and repeatability, with *k* < 3 as well. LOD can also be expressed in terms of surface density concentration of an analyte at saturation (g·mm^−2^), a condition typical of SPR-based optical platforms. Otherwise, one should talk about the minimum detectable concentration, the minimum value of the analyte concentration that the biosensor is able to detect reliably. This parameter, by not implying the utilization of the full calibration curve of the biosensor, requires only a few experimental points plus the reference point at 0 analyte concentration (i.e., blank measurement).
2. Specificity or selectivity, the sensor’s ability to be sensitive to a specific or a selective measurand (or target analyte, in the particular case of biosensors), instead of to all the other interfering measurands. As a general rule, one should first develop an experimental setup with controlled environmental conditions, both mechanical and thermal. After that, during the implementation of the bioassay, two options exist for performing the specificity test: (i) simple negative control, by injecting a solution containing (one or more) analytes different from the one under investigation; (ii) by spiking the analyte under investigation into a more real and complex solution that also containing other analytes (i.e., serum, plasma, blood, etc.). If the change in the sensor is within the sensor noise or, even so, lower than the change produced by the minimum detectable concentration, the specificity test is passed; otherwise, it has to be taken it into account.
3. Regeneration or reusability (percentage variation, %), a sensor’s ability to obtain the specific biolayer available for a new interaction in consecutive assay cycles, instead of for a single usage. The second kind of regeneration involves the restoration of the entire sensing layer, thus removing not only the bound analyte but also the immobilized bioreceptor and the functional layer. Clearly, for real applications, the first kind is the feasible one. To sum up, the steps of the regeneration test are the following: (i) choice of the best regeneration solution as a function of the analyte/bioreceptor pair used (each bioassay is usually regenerated by a specific regeneration solution); (ii) repetition (at least three times) of the measurement of the same analyte concentration after the respective cycle of regeneration.
